# Circular RNA-CDR1as acts as the sponge of microRNA-641 to promote osteoarthritis progression

**DOI:** 10.1186/s12950-020-0234-y

**Published:** 2020-02-18

**Authors:** Wei Zhang, Chi Zhang, Chengfang Hu, Congfeng Luo, Biao Zhong, Xiaowei Yu

**Affiliations:** 1 Department of Orthopaedics, Shanghai Sixth People’s Hospital East Affiliated to Shanghai University of Medicine & Health Sciences, No. 222 3rd Huanhu Road (West), Shanghai, 201306 People’s Republic of China; 2 Department of Orthopaedics, Shanghai Jiao Tong University Affiliated Sixth People’s Hospital, Shanghai, 200233 People’s Republic of China

**Keywords:** circRNA-CDR1as, miR-641, Extracellular matrix, Osteoarthritis

## Abstract

**Background:**

The antisense cerebellar degenerative-related protein-1 (CDR1as) has been identified as a sponge for several microRNAs. MiR-641 has been shown to be downregulated in osteoarthritic human chondrocytes, but its regulation and function in osteoarthritis (OA) has not been reported.

**Methods:**

OA cartilage samples were obtained from the knee joints of 12 patients (8 males and 4 females at age of 57–73 years old) who underwent total knee arthroplasty. Normal articular cartilage samples were obtained from the knee joints of 10 trauma patients at age of 29–65 years old (6 males and 4 females). The levels of circRNA-CDR1as mRNA and miR-641 were examined by qRT-PCR and the contents of type II collagen (Col II), IL-6, MMP13 and GAPDH in chondrocytes were examined by Western blot.

**Results:**

In this study, we found that circRNA-CDR1as level was significantly upregulated in OA chondrocytes, and negatively related with that of miR-641. RNA pull down assay confirmed that circRNA-CDR1as directly targets to miR-641. Furthermore, downregulation of circRNA-CDR1as increased type II collagen level but reduced MMP13 and IL-6 contents, while these effects were partly reversed by down-regulation of miR-641.

**Conclusion:**

Overall, our results indicate that circRNA-CDR1as plays a crucial role in regulating OA progression via modulating extracellular matrix metabolism and inflammation via sponging miR-641 and provide a novel regulatory role of circRNA-CDR1as in OA.

## Background

Osteoarthritis (OA) is a common, painful, degenerative, debilitating joint disorder and the fastest growing cause of disability worldwide, currently affecting over 8 million people and probably over 17 million by the year of 2030 [1, 2] and leading to significant morbidity due to associated joint pain and stiffness [3]. It affects all joint tissues and generally is characterized by structural changes, including degradation of articular cartilage at moving joints such as the knee and hip, common locations of osteoarthritis, as well as pathological changes in subchondral bone and associated synovitis [4].

Articular cartilage contains only one type of cells, the highly specialized chondrocytes*,* which are sparsely distributed in a dense extracellular matrix (ECM) and play critical roles in the development, maintenance, and repair of the ECM. By contrast, ECM, which mainly consists of water, collagen and proteoglycans, could affect free movement of the joint and how articular cartilage to withstand loads, and interact with integrin-mediated attachments to maintain the homeostasis of this environment [5] and the phenotype of its surrounding chondrocytes [6]. The degradation of ECM in articular cartilage especially its main component collagen during osteoarthritis could lead to a loss of these functions and OA development and progression [3]. Collagen degradation is mediated by collagenases such as MMP-13, which has the highest specific activity against collagen involved in osteoarthritis [7] and is known to be involved in maintaining the ECM equilibrium of cartilage as well as implicated in chondrocyte differentiation [8–10].

Inflammatory cytokines are involved in altering chondrocyte metabolism and phenotype, and therefore play a role in affecting matrix production and structure by activating catabolic pathways. Among them, IL-6 has been found to up-regulate MMP-1 and MMP13 [11, 12] and is detected in the synovial fluid of OA joints [13] .

The recently discovered circular RNAs (circRNAs) have become a hot topic in the field of non-coding RNAs. These RNAs form closed loops by covalently binding their 3′ heads to 5′ tails together [14, 15]. Unlike linear RNAs, they are more resistant to digestion by RNases and have much longer half-life [16]. Recent studies have revealed that many circRNAs are quite conserved evolutionarily and involved in the initiation and development of human diseases including OA [17–21]. Among these circRNAs, the antisense cerebellar degenerative-related protein-1 (CDR1as) has been identified as a sponge for several microRNAs [14, 17, 19, 22]. It is reported that circRNA-CDR1as contains about 70 conserved binding sites for miR-7 [22], which is involved in the progress of cancers [23], Alzheimer’s disease [20], insulin secretion [18], myocardial Infarction [24] and osteoblastic differentiation of stem cells [25] by regulation various signaling pathways. CircRNA-CDR1as also contains binding sites for other micoRNAs and functions as their sponges [26, 27], including those known to play critical roles in bone development and chondrogenesis [17]. MiR-641 has been shown to be downregulated in osteoarthritic human chondrocytes [28], but its regulation and function in OA has not been reported. Therefore, in this study, we explored whether circCDR1as functions as the sponge of miR-641 regulating OA development via affecting MMP13 and IL-6 levels in chondrocytes, with the hope to provide a novel way for understanding the regulated role of circRNA-CDR1as in OA, meanwhile put forward a new insight to its latent use in therapeutics.

## Methods

### Collection of human cartilage and isolation of chondrocytes

OA cartilage samples were obtained from the knee joints of 12 patients (8 males and 4 females at age of 57–73 years old) who underwent total knee arthroplasty. Normal articular cartilage samples were obtained from the knee joints of 10 trauma patients at age of 29–65 years old (6 males and 4 females). All tissues were subjected to histological examination and graded based on the modified Mankin scale [29]. All participants or their families have provided written informed consent. The study was approved by the Human Ethics Committee of Shanghai Sixth People’s Hospital East Affiliated to Shanghai University of Medicine & Health Sciences (China) and performed following the approved guidelines.

Cartilage sections were aseptically collected from all participants, sliced in full thickness, ground with a scalpel, incubated for 15 min at 37 °C in phosphate-buffered saline (PBS) containing 1% trypsin and centrifuged to discard the supernatant without chondrocytes. The collected cartilage was washed three times to remove trypsin and incubated in PBS supplemented with 2 mg/L of type IV collagenase for 12–16 h at 37 °C. The obtained chondrocytes were washed with Dulbecco′s modified Eagle′s medium (DMEM) supplemented with penicillin (100 units/ml)-streptomycin (100 μg/ml) and collected by centrifugation for 10 min at 200 g. The chondrocytes were stained with 0.4% trypan blue and their number and viability were examined using a Neubauer chamber.

### Chondrocyte culture

The collected chondrocytes were maintained in a 25 cm^2^ flask containing DMEM, 100 units/ml penicillin, 100 μg/ml streptomycin, 1% glutamine and 10% fetal bovine serum at 37 °C in a cell culture incubator supplemented with 5% CO_2_.

### Quantitative real-time PCR

Total RNA was isolated from chondrocytes using TRIzol and converted into cDNA using random primers as instructed. The cDNA was then used for by stem-loop quantitative real-time PCR (qRT-PCR) assay using forward primer 5′-TCAACTGGCTCAATATCCATGTC-3′ and reverse primer 5′-ACCTTGACACA GGTGCCAT-3′ for circRNA-CDR1as mRNA, forward primer 5′-TTATACTCTCAC CATTTGGATC-3′ and reverse primer 5′-TGACAAGATTTTACATCAAGAA-3′ for miR-641, forward primer 5′-TTACAGACCCCAGGCAGGCACA-3′ and reverse primer 5′-TCCATCAGCGTCAACACCATCA-3′ for RUNX2, as well as forward primer 5′-TCAAGCAGAAGAGAGAGGAG-3′ and reverse primer 5′-CCGTAACA CATTTAGAAGCC-3′ for FGF-2. All experiments were performed three times in triplicates. The data were analyzed using 2^−ΔΔCt^ method after normalized to GAPDH (for circRNA-CDR1as and FGF-2) or U6 (for miR-641).

### RNA transfection

CircRNA-CDR1as was cloned into pcDNA3.1 to obtain plasmid p-circRNA-CDR1as for overexpress circRNA-CDR1as and verified by Sanger sequencing. Plasmid p-siRNA-CDR1as containing small interfering RNAs (siRNAs) against circRNA-CDR1as, and miR-641 inhibitor were from GenePharma (China). Lipofectamine 2000 (Invitrogen) was used in the transfections in this study as instructed.

### Pull down assay with biotinylated circRNA-CDR1as probe

The biotinylated circRNA-CDR1as probe was designed to specifically bind to the junction area of circRNA-CDR1as and a random oligo was used as control probe. About 3 μg of probes were incubated with ~ 1 × 10^7^ cells that had been washed with ice-cold PBS and lysed in a lysis buffer for 2 h at room temperature. The biotin-coupled RNA complex was then incubated with streptavidin magnetic beads for 4 h and separated from the supernatant on a magnetic stand. After washed five times with the lysis buffer, miRNAs in the circRNA-CDR1as complex were isolated using Trizol and analyzed by qRT-PCR.

### Capture of biotin-coupled miRNAs

Biotinylated miR-641 mimics and nonsense control (NC) were obtained from GenePharma, Shanghai, China. 50 μuM of these oligos were transected into ~ 2 × 10^6^ chondrocytes at 50% confluence. Cells were washed and harvested after 24 h of transfection. The lysates were incubated with 50 μl streptavidin magnetic beads that had been pre-washed for 2 h and blocked for 4 h on a rotator at 10 r/min to pull down the biotin-coupled RNA complex. After wash five times, RNAs in the complex were recovered from the beads using Trizol LS (Life Technology, USA) and the abundance of circRNA-CDR1as in the complex was evaluated using qRT-PCR.

### Western blot

Western blot was performed to examine the contents of type II collagen (Col II), IL-6, MMP13 and GAPDH in chondrocytes. In brief, proteins in chondrocytes were isolated using Radio Immunoprecipitation Assay (RIPA) buffer containing 1 mM phenylmethane sulfonylfluoride. After determining protein concentration by BCA kit, the same amount of proteins were separated on SDS-PAGE and transferred onto PVDF membranes (Millipore, Massachusetts, USA). The membranes were then immersed in 5% non-fat milk for 1 h, probed overnight with 1:500 diluted primary antibodies against Col II (sc-52,658), MMP13 (sc-30,073), IL-6 (sc-130,326), FGF-2 (sc-365,106), GAPDH (sc-32,233, Santa Cruz, California, USA), p-MEK1/2 S9121, and p-ERK1/2 S4370 (Cell Signaling Technology), respectively, at 4 °C. After wash, proteins on the membranes were revealed by ECL chemiluminescence reagent (Pierce, Rockford, IL, USA) after 2 h incubation with horse-radish peroxidase-conjugated secondary antibody (Santa Cruz, California, USA) at room temperature. The intensity of each band was quantified using Image J and normalized to loading control of GAPDH.

### Immunofluorescence analysis

After rinsed with PBS, the cultured chondrocytes were fixed with 4% paraformaldehyde at room temperature for 15 min. Cells were then blocked with goat serum and then incubated with 1:200 diluted anti-Col II and anti-MMP13 antibodies overnight at 4 °C. Subsequently, cells were labeled by incubating 1 h with fluorescein isothiocyanate–conjugated AffiniPure goat anti-rabbit IgG (1:100 dilution) and with Hoechst 33342 for 5 min, and observed under a confocal microscope.

### Luciferase reporter assay

The binding sites of miR-641 found at at 3’UTR region of FGF-2 and were according to TargetScan online prediction software. The wild-type (wt) and mutant-type (mut) miR-641 binding sites at 3′ UTR of FGF-2 were designed by Shanghai Jima Gene Co., Ltd. All sequences were constructed into pGL3 vector (Promega Corporation, Fitchburg, WI, USA). For luciferase assay performed in HEK-293 T, cells in 24-well plates were co-transfected with 200 ng/well luciferase reporter constructs along with 400 ng/well miR-641mimic or mimic control using Lipofectamine 2000. In addition, 5 ng/well SV-Renilla luciferase plasmid was served as the internal control. Cells were harvested at 24 h after transfection and the luciferase activity was detected using the Dual Luciferase Reporter Assay kit (Promega) according to the manufacturer’s instructions. 30 μL protein samples were analyzed in a luminometer and luciferase activities were normalized to Renilla luciferase activity.

### Statistical analysis

Data were expressed as mean ± SEM. The differences between two groups and among multiple groups were compared using T-test and analysis of variance. A *p* value < 0.05 was considered statistically significant.

## Results

### CircRNA-CDR1as is upregulated in chondrocytes of OA patients

**Fig. 1:**
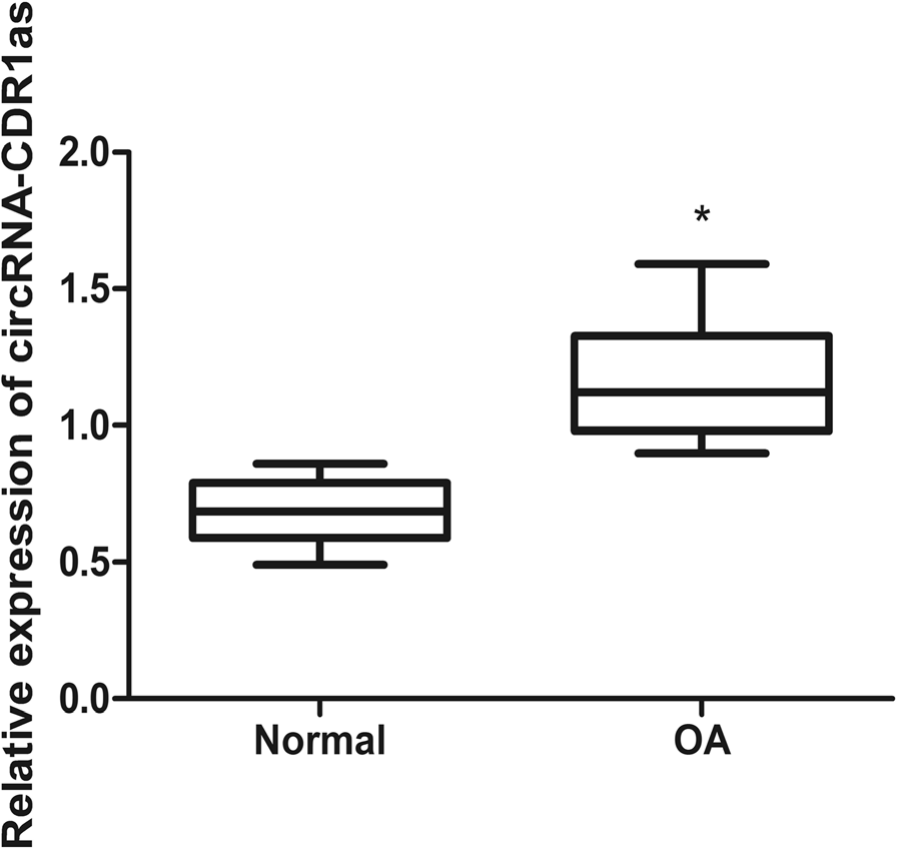
High circRNA-CDR1as expression in human OA chondrocytes. Shown are the representative results of qRT-PCR from at least 3 independent experiments, indicating that miR-641 level was upregulated in human OA chondrocytes. Data are expressed as mean + SEM. * *p* < 0.05 (t-test)

qRT-PCR examination of circRNA-CDR1as showed that circRNA-CDR1as level was significantly higher in OA chondrocytes than in normal chondrocytes (Fig. 1), indicating that circRNA-CDR1as may play a critical role in OA.

### Role of circRNA-CDR1as in catabolism and inflammation of ECM in chondrocytes

**Fig. 2:**
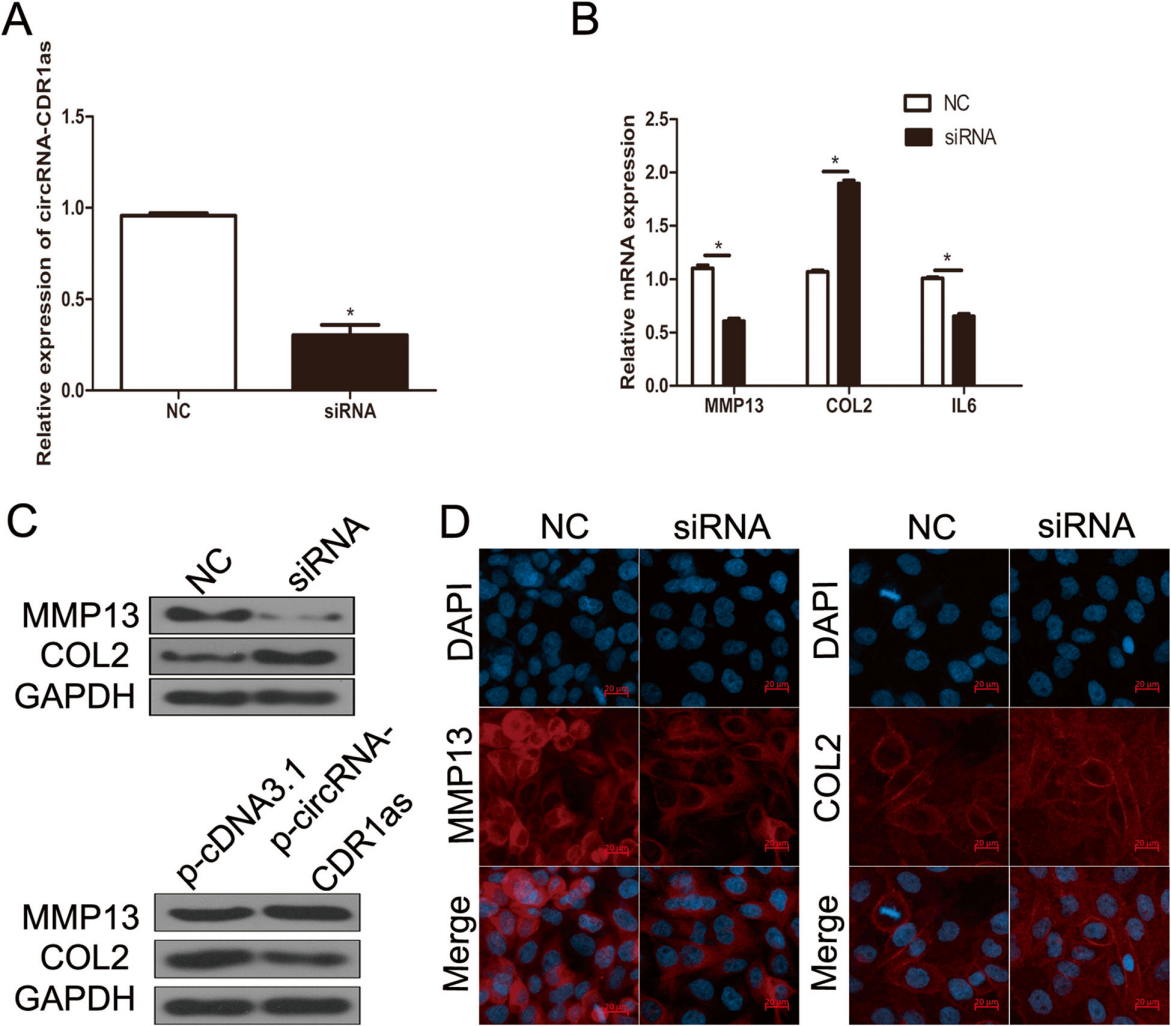
CircRNA-CDR1as play a role in human OA chondrocytes. **a** Normal chondrocytes were transfected with circRNA-CDR1as siRNA. The expression level of circRNA-CDR1as was analyzed by qRT-PCR. **b**-**c** OA chondrocytes were transfected with circRNA-CDR1as siRNA or p-circRNA-CDR1as. Col II, MMP13 and IL-6 levels were analyzed by qRT-PCR and western blot. **d** Following transfection of siRNA, expression of COL II and MMP13 were assessed by immunofluorescence. Scale bar, 100 μm. Data are expressed as mean + SEM. *p < 0.05 (t-test). Shown are the representative results from at least 3 independent experiments

qRT-PCR analysis valuating the effect of down-regulation of circRNA-CDR1as by siRNAs on the levels of inflammatory factors and ECM components in OA chondrocytes showed that compared with negative siRNA, transfection of circRNA-CDR1as siRNA significantly reduced circRNA-CDR1as level in OA chondrocytes (Fig. 2a). Moreover, Western blot, qRT-PCR and immunofluorescence staining all showed that siRNA-mediated downregulation of circRNA-CDR1as significantly increased Col II level, but reduced MMP13 level in OA chondrocytes (Fig. 2b-d). By contrast, overexpression of circRNA-CDR1as significantly enhanced MMP13 but reduced Col II expression at protein level (Fig. 2c). Furthermore, downregulation of circRNA-CDR1as markedly reduced the level of inflammatory factor IL-6 (Fig. 2b). Overall, these data showed that downregulation of circRNA-CDR1as is protective in OA against ECM degradation and inflammatory factor production.

### MiR-641 was a target gene of circRNA-CDR1as

**Fig. 3:**
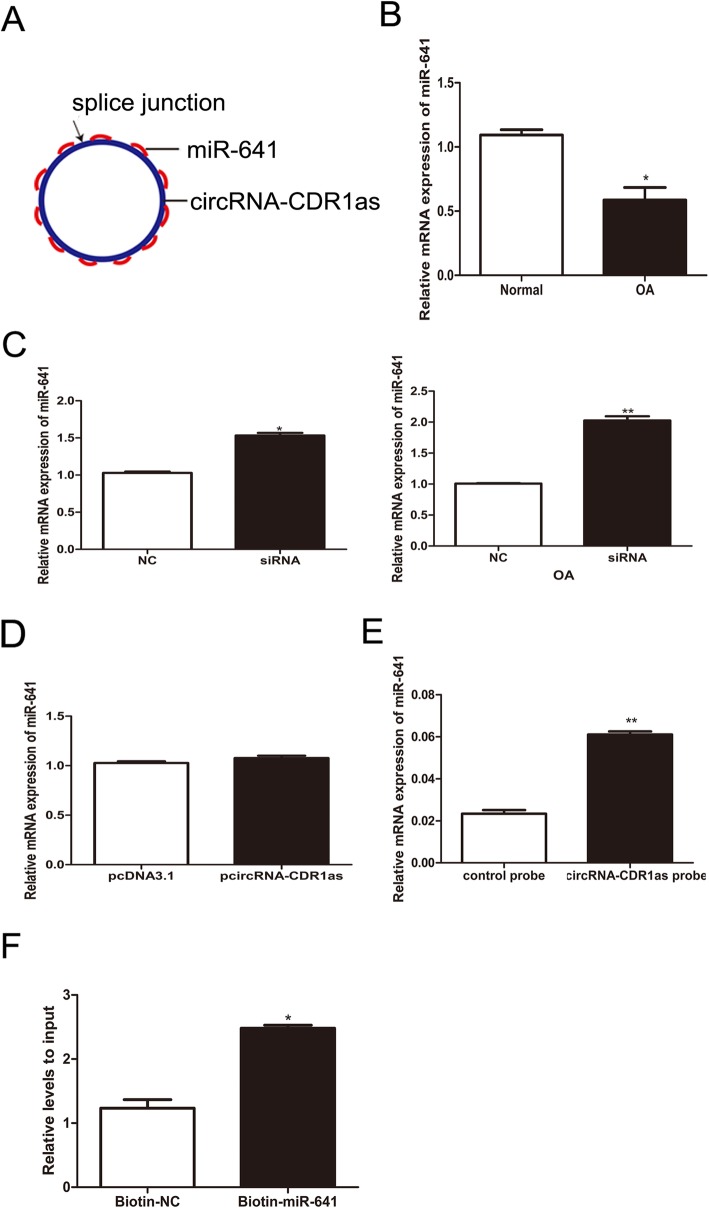
CircRNA-CDR1as interacts with miR-641. **a** circRNA-CDR1as contains 19 sites complementary to miR-641, as analyzed by the bioinformatics program starBase. **b** Detection of miR-641 by qRT-PCR. **c** Expression levels of miR-641 were assessed after knocking down circRNA-CDR1as. **d** Following transfection with p-circRNA CDR1as, the expression levels of miR-641 were detected using qRT-PCR. **e** miR-641was pulled down and enriched with circRNA-CDR1as specific probe and then detected by qRT-PCR. **f** The biotinylated miR-641 or its control is transfected into chondrocytes. After streptavidin capture, circRNA-CDR1as mRNA levels are quantified by qRT-PCR. Data are expressed as mean + SEM. *p < 0.05 (t-test). Shown are the representative results from at least 3 independent experiments

Increasing evidences suggest that circRNAs regulates miRNA target genes by acting as miRNA sponges. Thus, we hypothesized that circRNA-CDR1as could also target a specific miRNA and modulate its downstream functions. Therefore, we profiled the public database, starBase, and found that circRNA-CDR1as possesses 19 binding sites for miR-641 (Fig. 3a, Additional file 1: Figure S1), suggesting that miR-641 might be a target gene of circRNA-CDR1as. Further examination of the correlation between circRNA-CDR1as and miR-641 found that miR-641 level was reduced in OA chondrocytes and negatively associated with the level of circRNA-CDR1as (Fig. 3b). Moreover, siRNA-mediated knockdown of circRNA-CDR1as elevated miR-641 expression in normal chondrocytes or OA chondrocytes (Fig. 3c), while overexpression of circRNA-CDR1as by transfection of p-circRNA-CDR1as had no effect on the expression of miR-641 expression in normal chondrocytes (Fig. 3d). Studies have shown that circRNA-CDR1as is an abundant, largely cytoplasmic RNA. To assess whether circRNA-CDR1as could act as a sponge for miR-641, we performed the pull down assay using biotinylated circRNA-CDR1as probe in OA chondrocytes and observed that more miR-641 was detected in the circRNA-CDR1as probe-captured fraction than in the negative control probe-captured fraction (Fig. 3e). Additionally, biotin-coupled miR-641 captured more circRNA-CDR1as than biotin-coupled negative control, indicating that miR-641 could bind to circRNA-CDR1as (Fig. 3f). Taken together, these results demonstrate that circRNA-CDR1as could direct act as a sponge of miR-641.

### CircRNA-CDR1as participates in OA progression via targeting miR-641 in OA chondrocytes

**Fig. 4:**
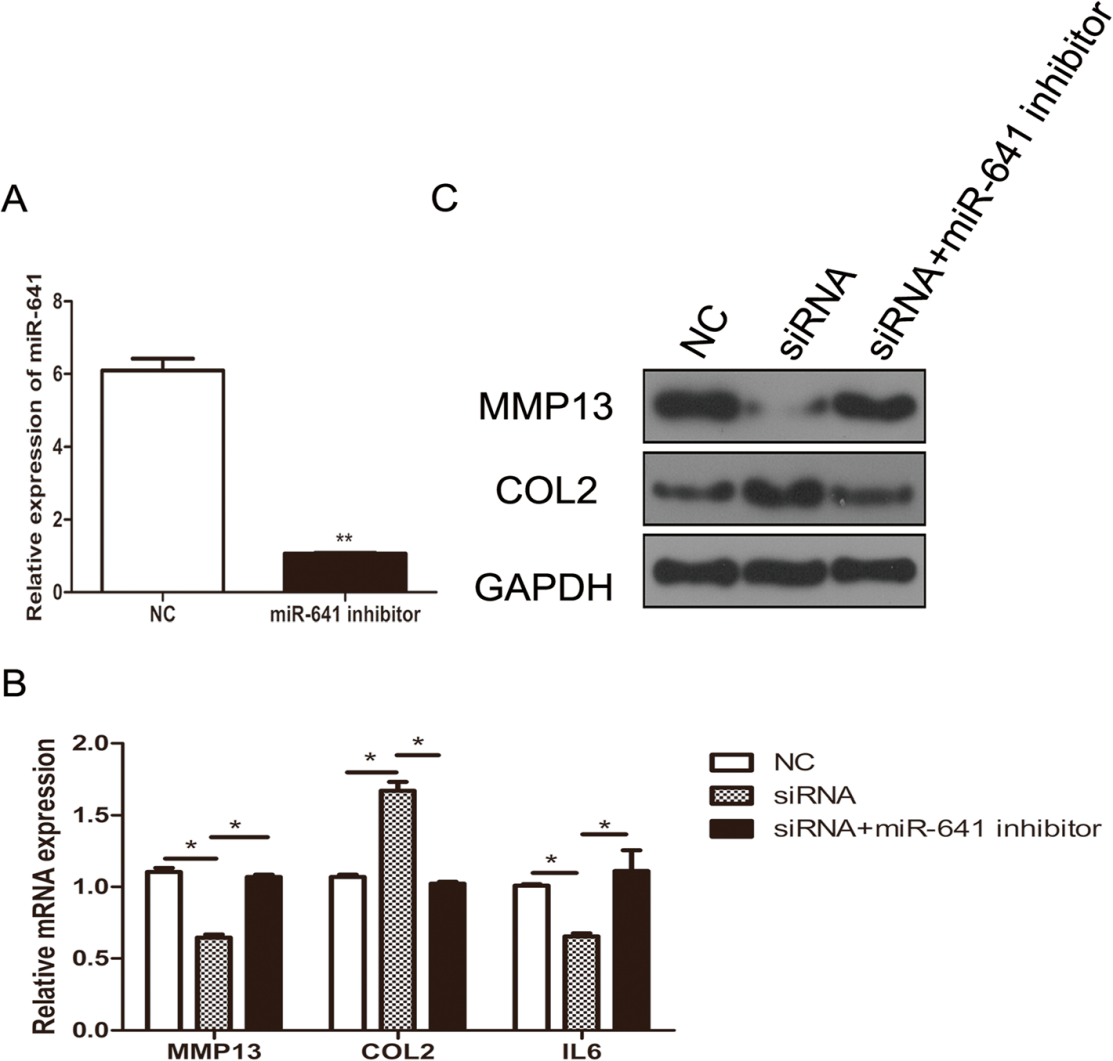
circRNA-CDR1as regulates the level of type II collagen, MMP13 and IL-6. **a** Changes in miR-641 abundance upon transfection with miR-641 inhibitors. MiR-641 abundance was determined by qRT-PCR. **b**-**c** The levels of MMP13, Col II and IL-6 were detected following transfection with circRNA-CDR1as, co-transfection with circRNA-CDR1as and the miR-641 inhibitor in chondrocytes. **b** The mRNA levels of MMP13, Col II and IL-6 were assessed by qRT-PCR. **c** The levels of MMP13 and Col II proteins were detected by western blot. Data are expressed as mean + SEM (n = 3). *p < 0.05 (t-test)

To further investigate whether miR-641 is a mediator of circRNA-CDR1as in OA chondrocytes, si-circRNA-CDR1as alone or along with a miR-641 inhibitor were transfected into OA chondrocytes. The levels of Col II, MMP13 and IL-6 mRNA and protein were respectively measured by qRT-PCR and western blot. The results showed that co-transfection with the miR-641 inhibitor in OA chondrocytes reversed the upregulation of Col II (Fig. 4a) and reduction of MMP13 level by knockdown of circRNA-CDR1as (Fig. 4b, c). Moreover, in consistence with the results in Fig. 2b, down-regulation of circRNA-CDR1as reduced the level of inflammatory factor IL-6 in OA chondrocytes, and this decrease was also reversed by cotransfection of the miR-641 inhibitor (Fig. 4b, c). Overall, these results suggest that circRNA-CDR1as could regulate ECM synthesis and inflammation by regulating miR-641 in chondrocytes.

### MiR-641 regulates MMP-13 and col II in OA chondrocytes via FGF-2 mediated MEK/ERK signaling pathway

**Fig. 5:**
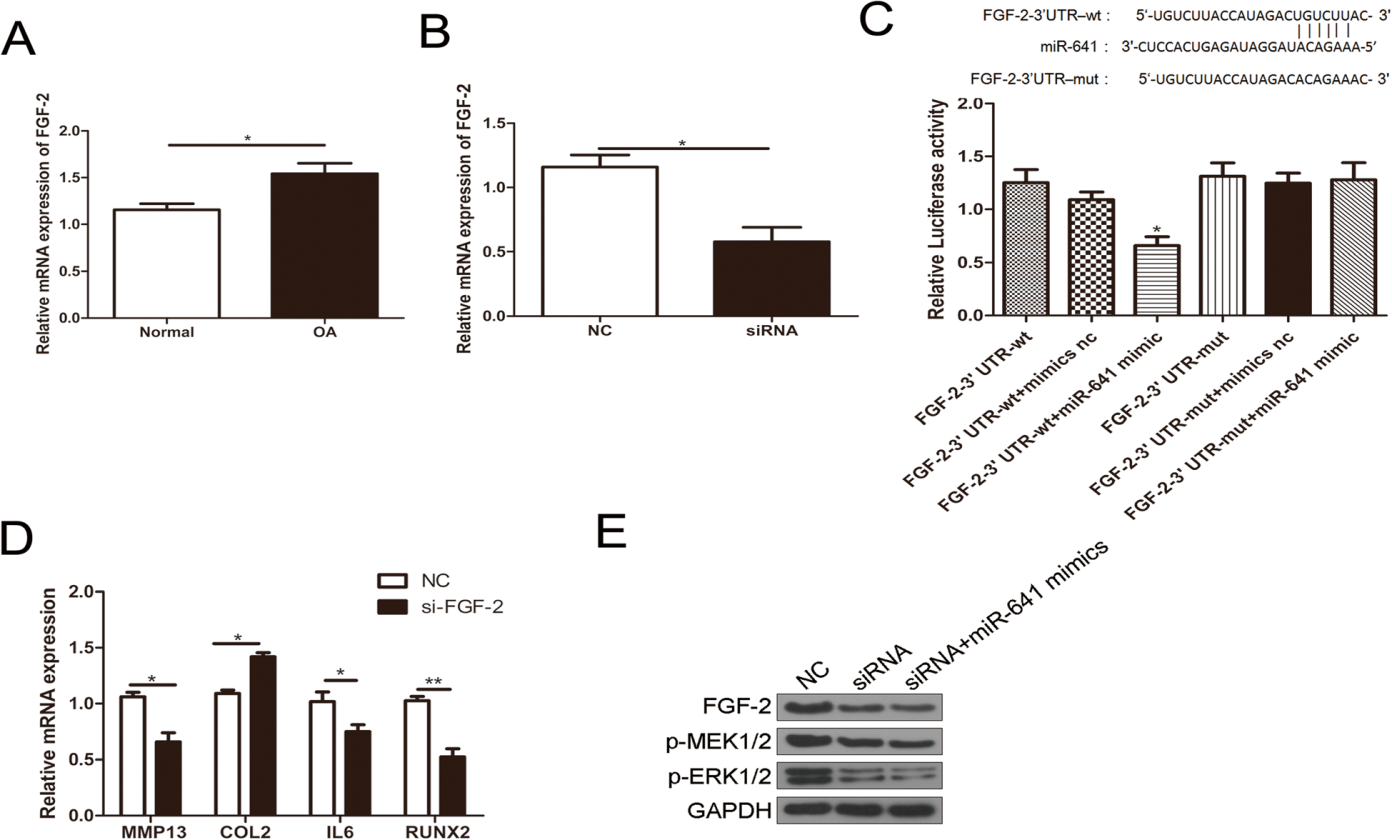
circRNA-CDR1as/miR-641/FGF-2 promotes the extracellular matrix metabolism and inflammatory process of OA chondrocytes by activating the MEK/ERK signaling pathway. **a** FGF-2 expression is upregulated in OA chondrocytes. **b** Upregulated FGF-2 in OA chondrocytes was abolished by circRNA-CDR1as siRNA. **c** Luciferase activity assay showed that the expression of FGF-2 was mediated by abolished in cells transfected with vectors containing 3′ UTR-wt of FGF-2 as well as miR-641 mimic. **d** Transfection of si-FGF-2 inhibited expression of MMP13, IL-6 and RUNX2, but enhanced expression of Col II. **e** Transfection of circRNA-CDR1as siRNA or cotransfected with miR-641 decreased the levels of FGF-2, p-MEK1/2 and p-ERK1/2

It has been reported that FGF-2 could up-regulate MMP13 expression by activating MEK/ERK signaling pathway, thereby enhancing RUNX2 activity [30]. To elucidate whether microRNA-641 regulating OA via FGF-2 mediated signaling pathway, we searched potential binding sites of miR-641 at 3′ UTR of FGF-2 on TargetScan website and found that 3′ UTR of FGF-2 contains 6 miR-641 binding sites. Therefore, we detected FGF-2 mRNA expression in chondrocytes from normal controls and OA patients using qRT-PCR. The results showed that FGF-2 expression was upregulated in OA chondrocytes and this upregulation was reversed by transfection of circRNA-CDR1as siRNA (Fig. 5a, b). Furthermore, we constructed dual luciferase reporter vectors with 3′ UTR-wt and 3′ UTR-mut of FGF-2 and found that the luciferase activity was significantly reduced in 3′ UTR-wt of FGF-2 plus miR-641 mimic group, but not in 3′ UTR-mut of FGF-2 plus miR-641 mimic group, indicating that miR-641 can directly bind to the 3′ UTR of FGF-2 (Fig. 5c). To determine whether FGF-2 is involved in extracellular matrix metabolism and inflammation of OA chondrocytes, we transfected FGF-2 siRNA into OA chondrocytes and found that MMP13, IL-6 and RUNX2 were downregulated, while Col II was upregulated (Fig. 5d). We found that knockdown of circRNA-CDR1as or cotransfected with miR-641mimics could downregulate the expression levels of FGF-2, p-MEK and p-ERK (Fig. 5e). Our results indicate that circRNA-CDR1as /miR-641/FGF-2 promotes the extracellular matrix metabolism and inflammatory process of OA chondrocytes by activating the MEK/ERK signaling pathway.

## Discussion

OA is a frequently occurring disease of joint, whose feature is degradation of articular cartilage at moving joints and pathological changes in subchondral bone. Much of the research on OA has focused on its diagnosis, pathogenesis, epigenetic regulation, and potential therapeutic purposes containing microRNAs. With the identification and further functional analysis of circRNA-CDR1as, its function as a sponge for multiple miRNAs has been revealed [14, 22–28]. But its role in OA development has not been explored previously. In this study, we showed that circRNA-CDR1as level was upregulated in human OA chondrocytes and down-regulation of circRNA-CDR1as by transfection of its specific siRNA in normal chondrocytes increased Col II level and reduced MMP13 and IL-6 levels, as confirmed by qRT-PCR, western blot and immunofluorescence staining, implying that circRNA-CDR1as shows protective effect in maintaining ECM homeostasis and regulation inflammation. CircRNA-CDR1as has been shown to play a critical role in regulation of tumor development, diabetics, and cardiovascular diseases [17–21]. To our best knowledge, this is the first report on its role in OA via regulating ECM homeostasis and inflammation.

CircRNA-CDR1as is known to act as sponge of multiple miRNAs. Besides miR-7, which has more than 70 binding sites in circRNA-CDR1as, circRNA-CDR1as has been reported to be sponge for miR-135a [31], MiR-1270 [26], and miR135b-5p [27]. In this study, we further expanded the family of its target miRNAs. Bioinformatic analysis found that circRNA-CDR1as possesses 19 binding sites for miR-641, a miRNA known differentially expressed in OA chondrocytes. qRT-PCR also showed that miR-641 level was negatively correlated with circRNA-CDR1as in OA chondrocytes and knock down of circRNA-CDR1as in normal chondrocytes enhanced miR-641 level. More importantly, RNA pull down assay demonstrated that circRNA-CDR1as and miR-641 were co-precipitated in normal chondrocytes. These data demonstrated that circRNA-CDR1as is a sponge for miR-641.

MiR-641 has been shown as a tumor suppressor by targeting MDM2 in human lung cancer [32], and a regulator of PI3K/Akt pathway in glioblastoma multiforme [33]. But its role in OA has not been previously reported. Given the role of circRNA-CDR1as in OA by regulating ECM homeostasis and inflammation, we hypothesized that miR-641 may involve in this process. Indeed, we found that treatment with miR-641 inhibitor reversed the effects of transfection of siRNA against circRNA-CDR1as in normal OA chondrocytes and the expression levels of Col II, MMP13 and IL-6 in OA the chondrocytes, which clearly indicated that circRNA-CDR1as regulates ECM homeostasis and inflammation by regulating miR-641 in chondrocytes.

A previous study has shown that FGF-2 regulates MMP-13 and Col II in OA chondrocytes via MEK/ERK signaling pathway [30]. Therefore, we elucidated whether miRNA-641 regulating OA via FGF-2 mediated MEK/ERK signaling pathway. In deed, we identified 6 miR-641 binding sites 3′ UTR of FGF-2 and showed that FGF-2 expression was up-regulated in OA chondrocytes and this up-regulation was reversed by transfection of circRNA-CDR1as siRNA via miR-641 (Fig. 5). Moreover, transfection of FGF-2 siRNA into OA chondrocytes up-regulated Col II, but down-regulated MMP13 and IL-6, as well as pMEK/ERK signaling pathway (Fig. 5), indicating that circRNA-CDR1as/miR-641/FGF-2 promotes the extracellular matrix metabolism and inflammatory process of OA chondrocytes by activating the MEK/ERK signaling pathway.

## Conclusion

Our study has clearly demonstrated that circRNA-CDR1as functions as a sponge of miR-641 to promote osteoarthritis progression in human by regulating ECM homeostasis and inflammation via FGF-2 mediated MEK/ERK signaling pathway and could be utilized as a biomarker and target for OA therapy.

## Supplementary information

**Additional file 1:Figure S1.** The analysis of potential miR-641 binding sites with circRNA-CDR1as. 19 potential binding sites of miR-641 for circRNA-CDR1as are listed.

## Data Availability

The datasets used and/or analyzed during the current study are available from the corresponding author on reasonable request.

## Abbreviations

CDR1as: Antisense cerebellar degenerative-related protein-1
circRNAs: circular RNAs
Col II: Type II collagen
DMEM: Dulbecco’s modified Eagle′s medium
ECM: Extracellular matrix
ERK: Extracellular signal-regulated kinases
FGF2: Fibroblast growth factor 2
MEK: Mitogen-activated protein kinase kinase
NC: Nonsense control
OA: Osteoarthritis
OA: Osteoarthritis
PBS: Phosphate-buffered saline
qRT-PCR: Quantitative real-time PCR
RIPA: Radio immunoprecipitation assay
