# Supplementary material for: Circular RNA-CDR1as acts as the sponge of microRNA-641 to promote osteoarthritis progression
Source: J Inflamm (Lond). 2020 Feb 18;17:8. doi: 10.1186/s12950-020-0234-y (PMC7029465; doi:10.1186/s12950-020-0234-y)
Supplement: Supplementary file 1 — Additional file 1:Figure S1. The analysis of potential miR-641 binding sites with circRNA-CDR1as. 19 potential binding sites of miR-641 for circRNA-CDR1as are listed. [file 12950_2020_234_MOESM1_ESM.docx]

**Supplementary Figure 1. The analysis of potential miR-641 binding sites with circRNA-CDR1as.** 19 potential binding sites of miR-641 for circRNA-CDR1as are listed.

**Target: 5' caacUGGCUCAAUAUCC-AUGUCUUc 3'**
**||:|||  ||||| |||||||**
**miRNA : 3' cuccACUGAG--AUAGGAUACAGAAa 5'**

**Target: 5' gucuuccagaaaauaUAUGUCUUc 3'**
**||||||||**
**miRNA : 3' cuccacugagauaggAUACAGAAa 5'**

**Target: 5' ugucuuccagcAUCUUCAUGUCUUc 3'**
**|||:| |||||||**
**miRNA : 3' cuccacugagaUAGGA-UACAGAAa 5'**

**Target: 5' gucuuccaUCAAAUUAAUGUCUUc 3'**
**|| | :: |||||||**
**miRNA : 3' cuccacugAGAUAGGAUACAGAAa 5'**

**Target: 5' aucuuccagCUAAUCCAUGUCUUc 3'**
**||| :| |||||||**
**miRNA : 3' cuccacugaGAUAGGAUACAGAAa 5'**

**Target: 5' gucuuccagCCAAUAUAUGUCUUc 3'**
**| | : ||||||||**
**miRNA : 3' cuccacugaGAUAGGAUACAGAAa 5'**

**Target: 5' ucuuccaacaaAUCC-AUGUCUUc 3'**
**|||| |||||||**
**miRNA : 3' cuccacugagaUAGGAUACAGAAa 5'**

**Target: 5' ucuuccaggaaAUCC-AUGUCUUc 3'**
**|||| |||||||**
**miRNA : 3' cuccacugagaUAGGAUACAGAAa 5'**

**Target: 5' gucuuccaaCAAAGCCAUGUCUUc 3'**
**| |  | |||||||**
**miRNA : 3' cuccacugaGAUAGGAUACAGAAa 5'**

**Target: 5' cuuccaAC-CAAGCC-AUGUCUUc 3'**
**|| | | || |||||||**
**miRNA : 3' cuccacUGAGAUAGGAUACAGAAa 5'**

**Target: 5' gucuuccaacaaaggUAUGUCUUc 3'**
**||||||||**
**miRNA : 3' cuccacugagauaggAUACAGAAa 5'**

**Target: 5' ucuuccagaCAAUCC-AUGUCUUc 3'**
**| |||| |||||||**
**miRNA : 3' cuccacugaGAUAGGAUACAGAAa 5'**

**Target: 5' ucuuccggaCAAUCC-AUGUCUUc 3'**
**| |||| |||||||**
**miRNA : 3' cuccacugaGAUAGGAUACAGAAa 5'**

**Target: 5' ucuuccaaaaaAGCC-AUGUCUUc 3'**
**| || |||||||**
**miRNA : 3' cuccacugagaUAGGAUACAGAAa 5'**

**Target: 5' ucuuccagaCUAUCC-AUGUCUUc 3'**
**|||||| |||||||**
**miRNA : 3' cuccacugaGAUAGGAUACAGAAa 5'**

**Target: 5' ucuuccagaaaAUCC-AUGUCUUc 3'**
**|||| |||||||**
**miRNA : 3' cuccacugagaUAGGAUACAGAAa 5'**

**Target: 5' ucuuccagaaaAUCC-AUGUCUUc 3'**
**|||| |||||||**
**miRNA : 3' cuccacugagaUAGGAUACAGAAa 5'**

**Target: 5' gucuuccaaCAAAGCCAUGUCUUc 3'**
**| |  | |||||||**
**miRNA : 3' cuccacugaGAUAGGAUACAGAAa 5'**

**Target: 5' ucuuccagaCUAUCC-AUGUCUUc 3'**
**|||||| |||||||**
**miRNA : 3' cuccacugaGAUAGGAUACAGAAa 5'**
